# Comparison of Object-Based Image Analysis Approaches to Mapping New Buildings in Accra, Ghana Using Multi-Temporal QuickBird Satellite Imagery

**DOI:** 10.3390/rs3122707

**Published:** 2011-12-16

**Authors:** Yu Hsin Tsai, Douglas Stow, John Weeks

**Affiliations:** Department of Geography, San Diego State University, 5500 Campanile Dr., San Diego, CA 92182, USA

**Keywords:** change detection, new building delineation, QuickBird, Feature Analyst, ENVI Feature Extraction

## Abstract

The goal of this study was to map and quantify the number of newly constructed buildings in Accra, Ghana between 2002 and 2010 based on high spatial resolution satellite image data. Two semi-automated feature detection approaches for detecting and mapping newly constructed buildings based on QuickBird very high spatial resolution satellite imagery were analyzed: (1) post-classification comparison; and (2) bi-temporal layerstack classification. Feature Analyst software based on a spatial contextual classifier and ENVI Feature Extraction that uses a true object-based image analysis approach of image segmentation and segment classification were evaluated. Final map products representing new building objects were compared and assessed for accuracy using two object-based accuracy measures, completeness and correctness. The bi-temporal layerstack method generated more accurate results compared to the post-classification comparison method due to less confusion with background objects. The spectral/spatial contextual approach (Feature Analyst) outperformed the true object-based feature delineation approach (ENVI Feature Extraction) due to its ability to more reliably delineate individual buildings of various sizes. Semi-automated, object-based detection followed by manual editing appears to be a reliable and efficient approach for detecting and enumerating new building objects. A bivariate regression analysis was performed using neighborhood-level estimates of new building density regressed on a census-derived measure of socio-economic status, yielding an inverse relationship with R^2^ = 0.31 (n = 27; p = 0.00). The primary utility of the new building delineation results is to support spatial analyses of land cover and land use and demographic change.

## 1. Introduction

Change detection is the process of identifying and quantifying temporal differences in the state of image pixels or objects through analysis of two or more registered image data sets [1]. Two strategies of image-to-image digital change detection are generally taken: (a) multi-temporal layerstack and (b) post-classification comparison [2]. Post-classification comparison change detection is one of the most commonly used change detection methods and the most straightforward strategy [2]. By classifying each image of a multi-temporal image set independently, analysts can produce change maps that represent a sequence of LCLU or round feature changes. Post-classification comparison holds promise because data from two dates are separately classified, thereby minimizing the problem of normalizing for atmospheric and sensor differences [1]. For object-based change detection, image objects or segments from an older image are compared to objects from a more recent image to delineate and identify land cover and land use (LCLU) change objects [2]. Multi-temporal layerstack change detection requires multiple dates of remotely sensed imagery be placed in a single dataset. For object-based change detection, image objects or segments derived from the temporal composite image are essentially temporal-spectral objects, which are subsequently classified to generate a thematic map of LCLU change and no-change objects [3]. While this method requires only a single classification, it is very complex, as change objects must be located for training and output maps represent change and no-change classes.

With the increasing number of satellite imagery types that have five meter or finer (higher) spatial resolution, more detailed information on landscape composition and dynamics can be derived. Commercial very high spatial resolution (VHSR) satellite images have many potential applications for image analysis, cartography, and photogrammetry including semi-automated delineation of urban structures and their changes over time [4]. VHSR remotely sensed imagery often possesses a high level of spatial and radiometric detail, which is efficient for visual interpretation and delineation [5]. However, visual interpretation methods may not be efficient or as standardized as more automated approaches to object delineation [6]. Several commercial software packages enable semi-automated image object recognition. Two object delineation approaches are tested in this paper—a spectral/spatial contextual approach (using Overwatch Geospatial Feature Analyst) and a true geographic object-based image analysis (OBIA) approach (using ENVI Feature Extraction).

Urban LCLU change information is required for a variety of applications including residential-industrial-commercial site selection, population estimation, tax assessment, development of zoning regulations, *etc*. [7,8]. Detecting and or delineating individual new buildings can provide useful information for urban planning, but is also challenging [9]. Buildings can be complex structures of various architectural details while appear similar in the visible wavelength [9]. Background materials or land cover types can range from paved and unpaved roads, various vegetation types like lawns and trees, and bare soil. Also, roof top facets can be differentially illuminated and cast shadows are captured with spatial resolutions finer than 5 m, which is generally required to delineate and identify complex urban features [10]. Up-to-date urban LCLU change information is especially needed for rapidly-growing developing countries. Unfortunately, LCLU change data in developing countries are commonly unreliable or non-existent [11].

Semi-automated, object-based image analysis has the potential to efficiently extract urban land cover and land use change information, such as the location and number of buildings constructed within a given time period. Much research has been conducted on extracting urban features in a semi-automated manner from VHSR image data [12–18], but little on detecting new building structures. Hurskainen and Pellikka [19] used an object-based approach to study the growth and change of informal settlements in Southeast Kenya using scanned aerial photography from three different dates. The post-classification comparison change detection method derived high accuracy in detecting and quantifying new buildings in the study area. Moeller and Blaschke [20] used QuickBird imagery from two dates to detect new buildings in the Phoenix, AZ metropolitan area. The authors found that principal components analysis performed best as an image enhancement for delineating new building structures. Matikainen *et al*. [21] utilized airborne laser scanner data and identified building changes using an automated OBIA approach. They concluded that the main problem of building change detection was false detection due to new or demolished buildings, and confusion with adjacent (background) features.

The objective of this paper is to determine how accurately new buildings in Accra, Ghana constructed in the 2002 to 2010 period can be detected and quantified using different OBIA procedures and change detection strategies applied to QuickBird multi-date satellite imagery. The paper summarizes one of the first attempts at directly detecting and delineating new buildings by combining OBIA with bi-temporal VHSR satellite data. In addition to testing the two object-based classification approaches/software, two image change detection approaches, post-classification comparison and bi-temporal layerstack classification were also evaluated. The image-derived maps representing new building objects were compared and analyzed based on a novel object-based accuracy assessment procedure. The density of new buildings was compared statistically against a measure of socio-economic status (SES) called the Housing Quality Index (HQI) [22] to test the hypothesis that greater building density was spatially associated with slum-like neighborhoods.

## 2. Data and Methods

### 2.1. Study Site and Data

Accra is the capital and largest city in Ghana with a population of almost four million people within the Greater Accra Metropolitan Area that spreads along the Atlantic coast near the confluence of the equator and prime meridian. With in-migration from rural areas, the city has expanded and become more densely settled in the last decade. The population of the Accra Metropolitan Area (our study site within the Greater Accra region) grew almost half million from 2000 to 2010. A majority of Accra’s inhabitants are poor and live in low SES neighborhoods. According to UN Habitat, 58% of the population lives in slum neighborhoods [23]. Most of these slums have informal, high density housing settlements with small vegetation coverage and high percentage of impervious land cover. The roofing materials of building within these settlements can be hard to distinguish from both impervious and unpaved backgrounds, even on VHSR imagery. Accra experiences two rainy seasons, one from May to July, and the other one from August to October. Higher SES neighborhoods are located at higher elevations where they are less prone to flooding from the short but intense tropical rains [24]. Houses in the higher SES neighborhoods are larger and less densely spaced, have more distinguishable rooftops, and are usually surrounded by paved and landscape vegetation.

**Figure 1:**
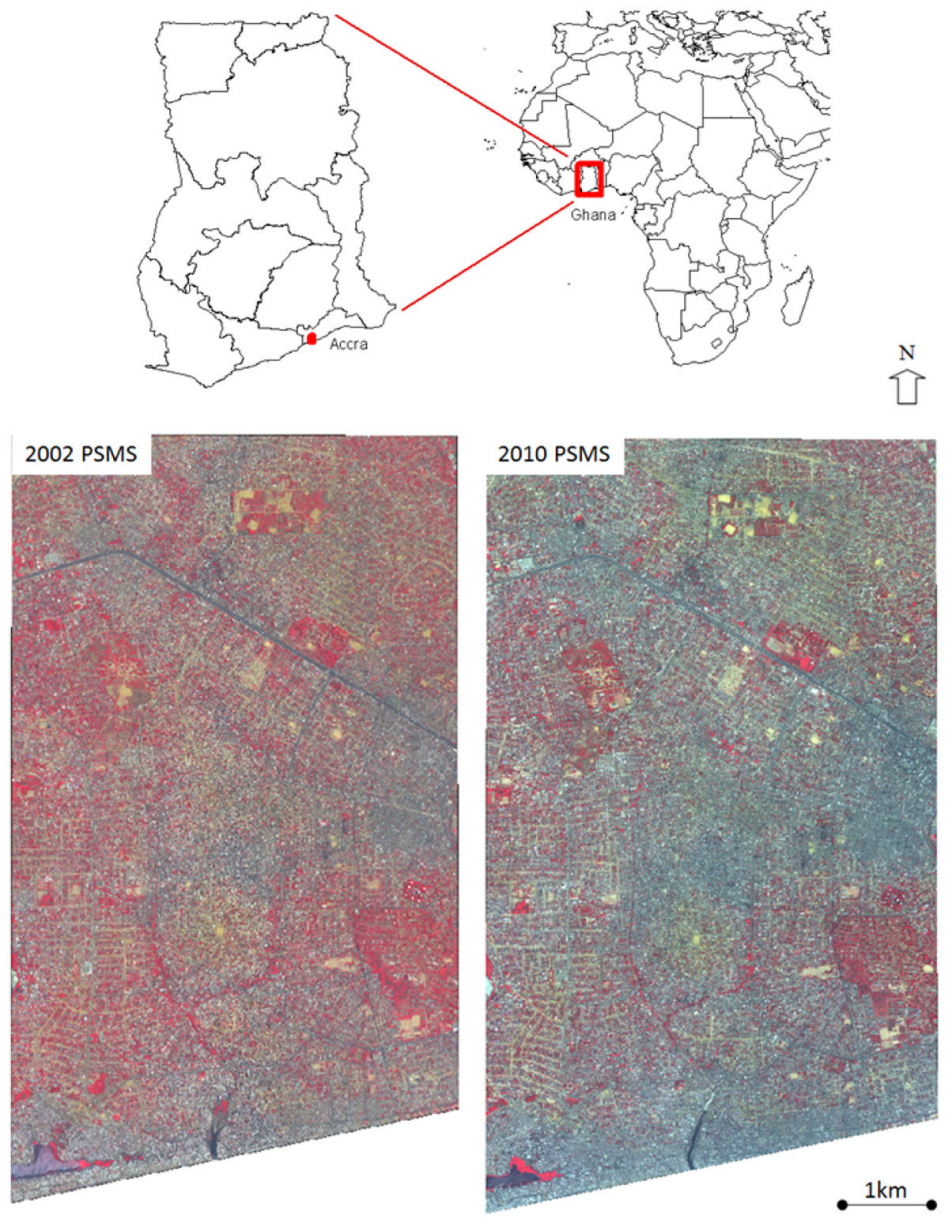
Map of Accra, Ghana. The study area for 2002 and 2010 QuickBird pan-sharpened multispectral images are displayed in standard false color infrared composite format.

QuickBird images used for this project were captured on 12 April 2002 and 18 January 2010. QuickBird is a commercial earth observation satellite which collects panchromatic (Pan) imagery at 0.6 m spatial resolution and multispectral (MS) imagery at 2.4 m resolutions. The two sets of imagery had very small (*i.e.*, 6.3°) view angle difference (18.9° for the 2002 image and 12.6° for the 2010 image) and a moderate (*i.e.*, 10.6°) solar zenith angle difference (21.3° for the 2002 image and 31.9° for the 2010 image). Each image was geometrically corrected separately by a third-party vendor. The 2010 dataset was geometrically registered to the 2002 dataset using a second-order polynomial warping transformation with 200 control points to ensure more accurate image registration and therefore, more accurate change detection results. The model fit of the warping transformation for the image-to-image registration was RMSE = 2.2 pixels. Radiometric normalization was performed using the pseudo-invariant feature approach [25,26] to align the 2010 image to the 2002 image to minimize the radiometric differences between the two QuickBird images. Figure 1 shows the subset of 2002 and 2010 pan-sharpened multispectral (PSMS) QuickBird images covering Accra that were used in this paper to test the change detection approaches and validate the results. The subset was delineated in an effort to provide balance between tractable processing times and data storage, and representation of most of the land use and SES types found throughout Accra. The pan-sharpening algorithm was based on a principal component transformation, with cubic convolution interpolation applied to artificially disaggregate the MS data. The subset is cloud- and cloud shadow-free and covers 35 km^2^ of the western portion of Accra.

### 2.2. New Building Delineation Approaches

Both post-classification comparison and bi-temporal layerstack methods were performed using Feature Analyst for ArcGIS and ENVI Feature Extraction software. All image-derived products were generated in vector format. The 2002 and 2010 PSMS image layers (Red, Green, and NIR wavebands for both dates) and image transforms were stacked to create a bi-temporal image set and used as the input for the bi-temporal change detection methods. Two types of image transforms were generated and included into the object delineation processing in an attempt to minimize shadow and differential and illumination effects and increase classification accuracy: (1) normalized difference vegetation index (*i.e.*, difference between the NIR band and the Red band divided by their sum: NDVI = (NIR − Red)/(NIR + Red)); and (2) normalized difference Red/Blue index [27] (*i.e.*, difference between the Red and the Blue band divided by their sum: NDRBI = (Red − Blue)/(Red + Blue)). The 2002 and 2010 image data sets (Red, Green, and NIR wavebands from PSMS images and derived normalized index layers) were classified separately in the post-classification comparison change detection method. The Blue waveband was not used in the change detection phase after visually determining that it did not provide additional information in delineating new buildings and has lower contrast due to haze effects. However, the Blue band is incorporated into the NDRBI.

Seven land-cover classes were utilized in the post-classification comparison method: bight rooftops, dark rooftops, vegetation, dark pavement, bright cement, bare soil, and shadow. Two primary transition classes (*i.e.*, new buildings and vegetation loss) were added to these seven classes when implementing the bi-temporal layerstack change detection method. The post-processing phase of the post-classification comparison method, including reclassification, map comparison, and masking, was performed with ArcGIS software in order to derive building change/no change maps. The single-year, seven-class LCLU maps were recoded into building and non-building classes and exported as a thematic map portraying building objects. Two final building thematic maps were created, one for each year. Map comparison was performed using these thematic maps from year 2002 and 2010. After the bi-temporal layerstack classification, the nine-class result was recoded into a two-class change map depicting “new buildings” and “other” land cover types. Two water bodies in the study area were masked out from the final products.

Feature Analyst is an adaptive-learning software package from Overwatch Geospatial (formerly Visual Learning Systems, Inc). We applied this tool for object recognition and surface feature extraction; the tool uses inductive learning algorithms and techniques to model visual object-recognition processes [28]. Spectral and local spatial information from images were incorporated to classify individual pixels [29] based on target and background signatures. Pre-defined search kernel patterns were selected, as well as input image bands. The software implements a proprietary machine learning algorithm modeled after the human visual system for image interpretation [28]. Object extraction was accomplished by quantifying visual image interpretation parameters for building and background features and training machine learning components with parameters [29].

For the Feature Analyst spectral/spatial contextual machine learning approach evenly distributed training samples were visually identified and digitized for all the training classes based on the PSMS images. Table 1 shows the number of training samples for the machine learning algorithm. Machine learning parameters were selected to control the building delineation process. Learning parameters, including the search kernel patterns/sizes and minimal object sizes were explored interactively before the image classification/building delineation process to derive products that best represent the imagery. The search kernel patterns that were tested are square, Manhattan (*i.e.*, a diamond shape), and various types of Bull’s Eye (*i.e.*, focusing on the center and edges of moving windows). Kernel sizes of 3 by 3 pixels, 5 by 5 pixels, 7 by 7 pixels, and 9 by 9 pixels were tested. Various values ranging between 5 and 40 pixels representing the minimal derived object size were also explored interactively. Different learning parameters and kernels were utilized for processing of single date and bi-temporal composite data sets.

Feature Extraction, a module of the ENVI image processing software package from ITT Visual Information Solution (formerly Research Systems, Inc.), implements a standard OBIA approach consisting of segmentation, segment-classification and generalization, based on attributes of spatial, spectral (brightness and color), and texture characteristics [30]. We utilized Feature Extraction to segment QuickBird images into regions of pixels, computed attributes for each region to create objects, and classified the objects (with a supervised nearest neighbor classifier to delineate new building objects).

The scale level, a parameter of Feature Extraction that controls relative segment size, was manipulated interactively to optimize the segmentation process. The segmentation size was adjusted to be able to represent the minimum-sized feature of interest (e.g., smaller buildings). The merge level (*i.e.*, a parameter for merging contiguous segments based on a homogeneity criterion) was determined after the segmentation to group overly small and contiguous segments into larger objects. Based on testing parameters interactively, a small segmentation size (e.g., 25 on a scale from 0 to 100) followed by a large merge level (e.g., 75 on a scale from 0 to 100) created objects that best represent the majority of buildings in the study area. Evenly-distributed training samples were selected for each classification category based on the previously segmented and merged objects for performing a supervised classification based on a *K-nearest neighbor* classifier. The K parameter is the number of neighbors considered during classification [30]. Table 1 shows the number of training objects used for classification. The same segmentation, merge and classification parameters were utilized for processing of both single date and bi-temporal composite data sets.

### 2.3. Accuracy Assessment

**Figure 2:**
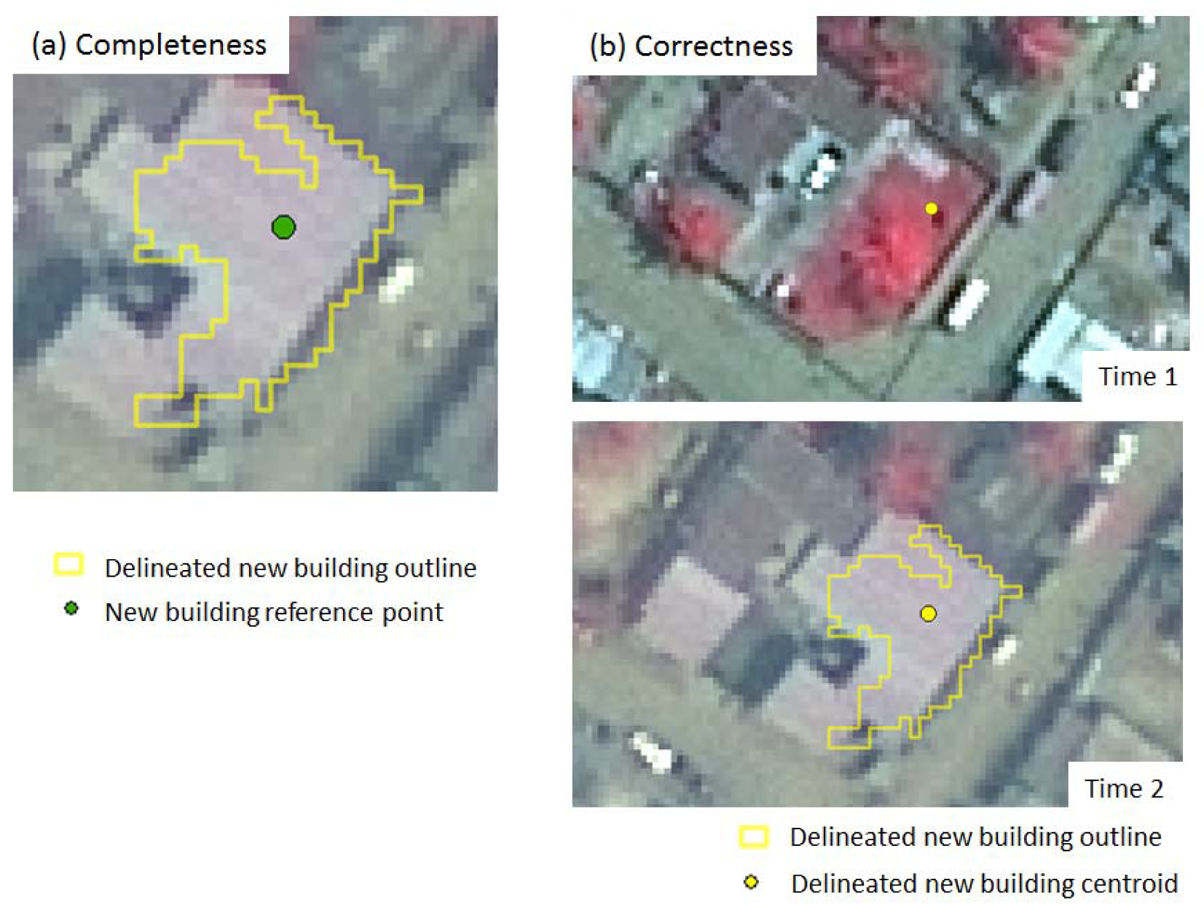
Accuracy assessment of delineated new building maps. The assessment consisted of calculating **(a)** completeness and **(b)** correctness values. Completeness quantifies the matched percentage between delineated new building polygons and new building reference points. Correctness is the percentage of matching between delineated new building points and pan-sharpened multispectral (PSMS) reference images. Time 1 = 12 April 2002. Time 2 = 18 January 2010.

Image-derived building change maps were subjected to an object-based accuracy assessment. Lippitt *et al*. [31] demonstrated that the sizes of OBIA derived segments are smaller than reference polygons used for accuracy assessment. This can lead to a high omission error. The focus of this paper is on the location, number and density of new buildings, rather than on how accurately the shapes or sizes of new buildings are represented. Object-based accuracy assessment was conducted using ArcGIS software. Figure 2 shows the assessment of building change maps based on two point-in-polygon measures: *completeness* (*i.e.* number of matched reference objects/number of reference objects) and *correctness* (*i.e.* number of matched extraction objects/number of extracted objects) of change features as described below [32]. Change features that were smaller than a threshold of 25 m^2^ were considered classification noise and removed from change maps and therefore, the accuracy assessment as well.

In order to test the completeness of new building detection, reference points that represent new buildings were manually digitized based on visual interpretation of the PSMS imagery. One hundred four (104) reference points of new buildings were randomly selected from several clusters of points drawn from three Enumeration Areas (EAs), the finest geographic level of census reporting unit in Accra. These EAs were selected to represent different levels of SES based on demographic census data. An additional 96 reference points distributed evenly in the study area (*i.e.*, systematic spatial sample) were also selected and visually interpreted. In order to create the dispersed set of reference points, a grid layer that equally divides the study area into 96 rectangles was overlaid on the PSMS imagery. One new building object was identified and digitized within each rectangle. All 200 reference points were overlaid and compared to the extracted change feature polygons. If a reference point was located within a delineated new building polygon as Figure 2(a) shows, it was recorded as correctly identified.

To test for correctness, 200 new buildings delineated by OBIA were randomly selected and compared to the reference images. Centroids of image-derived new building polygons were extracted to represent the change feature locations. The centroids were visually compared to the QuickBird PSMS images to verify if the point represented an actual new building (see Figure 2(b)). The completeness and correctness of change detection results were calculated to assess the performance of different feature delineation methods and different change detection approaches.

In order to further explore the reliability and utility of new building products, the delineated new building map derived from the post-classification comparison method using spectral/spatial contextual approach which contained a sufficient amount of features to modify was edited manually through visual interpretation of PSMS imagery. Based on experimentation, deleting false objects was more efficient than digitizing omitted new building objects. Incorrect building change objects generated through the classification and map comparison phase were removed during the editing process. Objects that were delineated as new buildings but were not detected during the automated feature delineation process were manually digitized as new buildings. The numbers of features that were deleted and added were recorded, as was the total new building features.

### 2.4. Delineated New Buildings and Socio-Economic Status

The new building map derived from OBIA and subsequent manual editing was used to generate a new building density map and evaluated statistically with the SES data. A housing quality index (HQI) was used to represent the SES of an aggregated neighborhood unit called “vernacular neighborhoods” [33]. Vernacular neighborhoods refer to neighborhood boundaries that are broadly recognized and agreed by residents of Accra. The HQI was created from a principal components analysis using a set of variables for all characteristics of housing and infrastructure available from the Ghana 2000 census at the housing unit level [22]. The variables included type of floor/toilet, availability of electricity/water, kitchen facility, and way of disposing liquid and solid waste. A value of zero indicates the poorest housing quality and a value close to 5 (the maximum of HQI) indicates the highest housing quality. The HQI ranges from 1.70 to 3.77 in the study area. The number of delineated new buildings for each neighborhood was divided by the neighborhood area to create the new building density map. A bivariate regression was performed to assess the strength of relationship between the density of new buildings as a function of HQI.

## 3. Results

**Figure 3:**
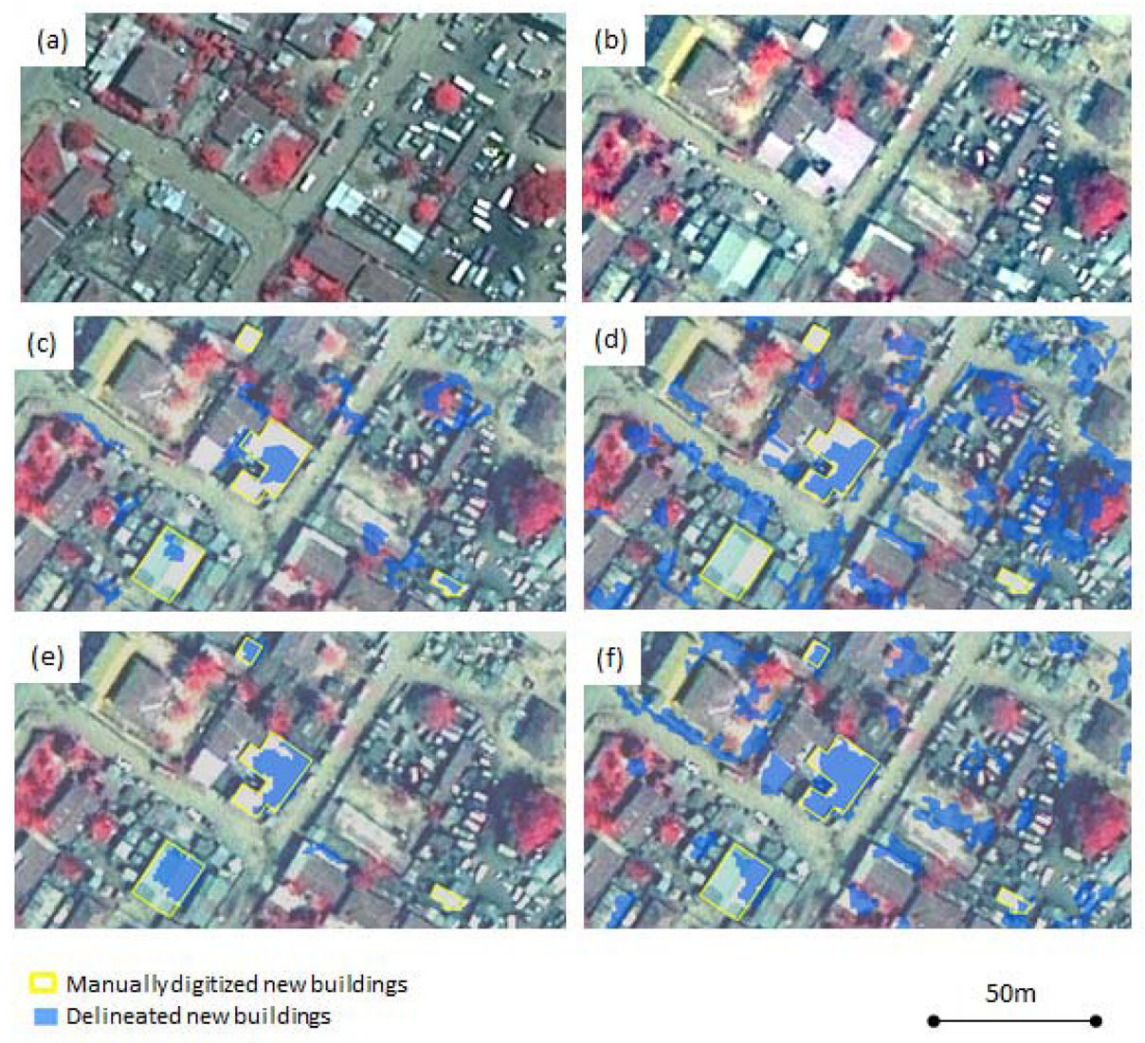
A subset of delineated new results. **(a)** 2002 pan-sharpened multispectral (PSMS) image subset. **(b)** 2010 PSMS image subset. **(c)** Post-classification comparison method using Feature Analyst had a few misclassified objects. **(d)** Post-classification comparison method using ENVI Feature Extraction had many misclassified errors. **(e)** Bi-temporal layerstack method using Feature Analyst had little noise. **(f)** Bi-temporal layerstack method using ENVI Feature Extraction had many false delineations.

On average for the new building identification products, over 36,000 new building objects were delineated within the approximately 35 km^2^ study area; three of the four products contained over 40,000 new building objects, as shown in Table 3. Of the four semi-automated new building delineation maps, the bi-temporal layerstack method using the spectral/spatial contextual approach generated the smallest number of new building features. Figure 3 shows a subset of the delineated new building results. Besides replacing vegetation or other structures, a high percentage of new buildings had been constructed within empty spaces (usually bare soil or concrete) between other housing structures. As is evident in Figure 3(d,f), the maps derived from the object-based feature delineation approach depict the greatest number of new building features. Many false delineations of new building objects resulted from this approach. Large newly constructed structures (*i.e.*, more than 350 m^2^) were mostly industrial or public buildings. Residential structures ranged from 30 to 300 m^2^ in area. A greater number of smaller new buildings were delineated in low SES areas than other areas.

Different kernel patterns and sizes were tested in a trial and error manner using the preview function in Feature Analyst in order to generate maps that best delineated buildings selected for training. The image inputs for this approach were each classified using a different set of parameters. Table 4 shows that the 2010 data set in the post-classification comparison method was classified using the smallest (3 × 3) search kernel among the three datasets; the largest (93 × 9) kernel size was used in the bi-temporal layerstack method. In general, a *Bull’s Eye* kernel pattern worked well as it incorporates both the central area surrounding the pixel (primarily “target”) being classified and a symmetrical zone surrounding the central area (primarily “background”) into account when performing classification. Building sizes and the proximity between adjacent buildings were the main factors for testing different classification parameters. The 2010 image appears to contain more small buildings and has less distinct object boundaries (apparently from haze-related contrast reduction) which required the use of the smallest kernel size. A larger kernel that focused on the center and corners was required to capture building changes from the bi-temporal stacked image dataset. Selecting kernel sizes that represent the majority of building features was more effective than using different kernel patterns. The post-classification comparison method delineated many more new buildings than the bi-temporal layerstack method, as previously noted and shown in Table 3. Figure 3(c,e) also illustrates that the bi-temporal layerstack method generated fewer new building objects than the post-classification comparison method. However, the post-classification comparison method yielded slightly higher completeness values and the bi-temporal layerstack method yielded higher correctness values, as shown in Table 4.

The segmentation scale level, merge level, and K classification parameter for Feature Extraction were all manipulated to optimize the building change results by using the preview function. Table 5 indicates that all image inputs were segmented using different scale levels and the objects were later merged using different merge levels. The optimal segmentation scale was determined by delineating the proper shape of buildings from their backgrounds. The final merge level was determined by generating the fewest number of segments that represented individual buildings. With 2010 image contained more small buildings, the smallest segmentation level was applied in order to create smaller objects. The bi-temporal layerstack image had a smaller merge level to preserve various sizes of buildings from both dates. A K parameter of 3 yielded relatively accurate classification maps compared to parameters of 1, 5, or 7. The K parameter can drastically change the classification results.

Large K parameters created overly generalized results while a K parameter of 1 created obvious false detections. All attribute information (spatial, spectral, and texture) was incorporated when classifying the 2002 and bi-temporal stack datasets. All attributes except area were used when classifying the 2010 dataset to reduce the over-classifying issue. The object-based feature delineation approach generated the largest number of new building features (see Table 3). Both post-classification comparison method and bi-temporal layerstack method change detection strategies using the object-based feature delineation (*i.e.*, Feature Extraction software) approach yielded lower correctness than completeness value (see Table 5) indicating an over-mapping of building objects. This approach yielded many false delineations.

After careful visual comparison of all new building products, the product from the post-classification comparison method using the spatial contextual approach was determined to capture more of the building change objects (*i.e.*, minimal omissions) for manual editing and product refinement. The final edited product contained 11,747 new buildings, or approximately 340 new buildings constructed per km^2^ during the study period. More than 80% of the originally delineated objects (33,198 out of 40,426) were deleted and 4,519 new buildings were added during the editing process. A majority of the objects that were originally omitted and subsequently added through the manual editing were buildings with bright reflective rooftops. For the final edited product, 62% of the objects were generated through the automated feature delineation approach; 38% of the map were manually identified and added.

The relationship between new building density (derived from the manually edited product) and HQI was explored using a bivariate regression analysis at the vernacular neighborhood level. This enables the hypothesis that greater building density was spatially associated with slum-like neighborhoods to be tested. The assumption is that HQI affects the number of new buildings as neighborhood-level patterns of health can be accounted for by using two broad categories of predictor variables: poverty (who) and place (where), and these patterns can be discerned from the analysis of high spatial resolution satellite imagery. Our goal is to accurately delineate new buildings in Accra for use as an indicator of LCLU and population change and their effects on social phenomena relating to health outcomes.

**Figure 4:**
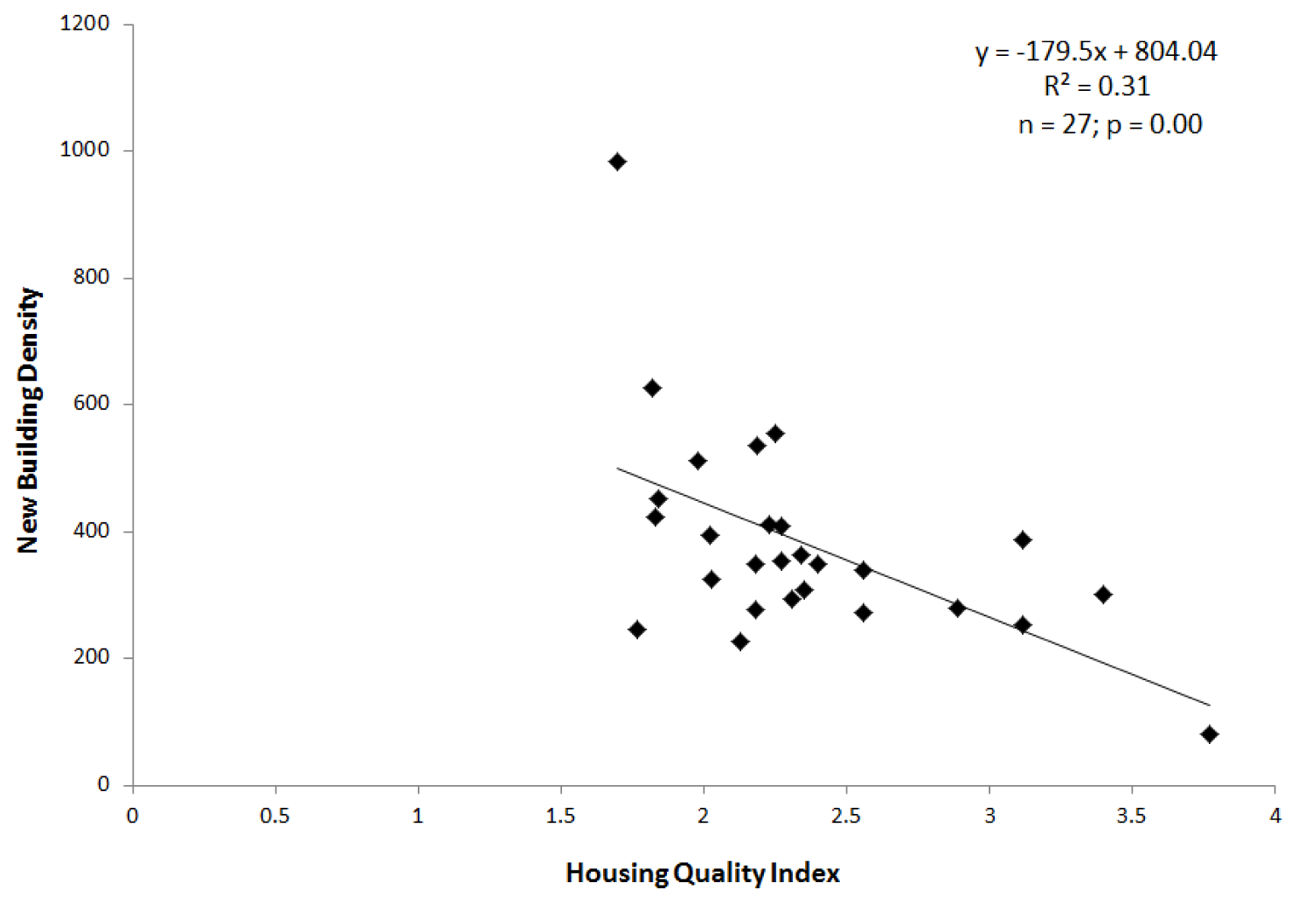
Scatterplot and regression results of new building density *vs.* Housing Quality Index (HQI).

**Figure 5:**
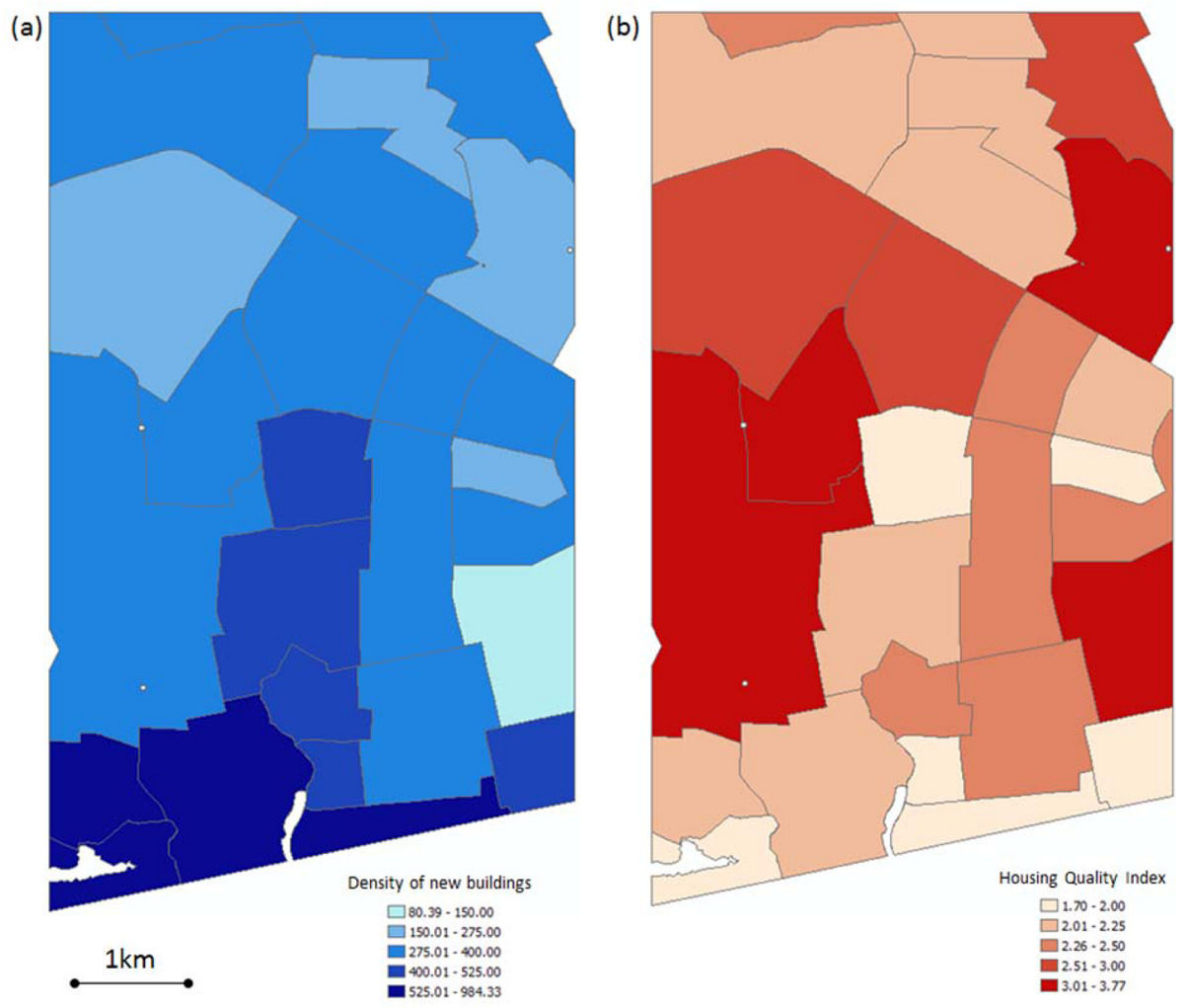
Neighborhood-level maps of **(a)** new building density map derived from manual edited post-classification comparison and spectral contextual approach product and **(b)** Housing Quality Index (HQI) map.

A significant inverse relationship (R^2^ = 0.31; n = 27; p = 0.00) was determined, as shown in Figure 4. The scatterplot shows that the while the regression coefficient is significant, the relationship between new building density and SES is not particularly strong. When estimating building density from the same image-derived product but without manual editing an R^2^ value of 0.10 (n = 27; p = 0.10) resulted. An unusually high percentage of small new buildings had been constructed within empty spaces between other housing structures at the southwest coastal area and therefore created a neighborhood outlier with extremely high new building density. Efforts were made to remove the extreme value as shown in Figure 4. The outlier had minimal (*i.e.*, 0.0005 difference on the R^2^ value) influence on the regression results so the value was kept in the analysis. Based on visual analysis of the neighborhood-level maps of new building density and HQI, hot spots of new building construction tended to be located in the coastal and central portions of the study area and appear to mostly correspond to low SES regions as seen in Figure 5.

## 4. Discussion

In general, the bi-temporal layerstack approach to change detection yielded more accurate and reliable maps of new building objects than the post-classification comparison method, as did the spatial contextual machine learning classification method (Feature Analyst) relative to the true OBIA (Feature Extraction) method. None of the resultant products from these semi-automated routines were sufficiently accurate and reliable to be of direct utility for urban planning or scientific analyses of LCLU change or demographic analyses, without some form of manual editing and revision, at least not in this setting of a city in a developing country. However, this is normally the case for most attempts at automation in image processing and photogrammetry (except for simplistic image analysis tasks), such that derived maps are not sufficiently accurate without considerable manual editing.

Confusion between roof materials and adjacent background land covers, and shadow effects were the main sources of error, both in terms of excessive commission and in a few cases omission errors. Although ‘shadow’ was one of the classification categories, buildings casting shadows of different lengths and directions between the two dates of imagery frequently caused misclassifications of building changes (mostly false positives). Change objects representing changing material types (particularly new highly reflective roof materials) rather than building construction changes, such as from replacement of rooftop materials and new pavement areas, often caused misclassification. Also, bright soil patches that are commonly adjacent to buildings were often misclassified as buildings. Another type of confusion was between buildings and bright shipping containers. Large shipping containers were commonly seen in the study area and their size was greater than minimum 25 m^2^ object size threshold. The confusion between new buildings and shipping containers was common for both spatial/contextual and object-based approaches. Some vehicles were delineated as new buildings, but they were generally small enough to be excluded from the final new building maps.

**Figure 6:**
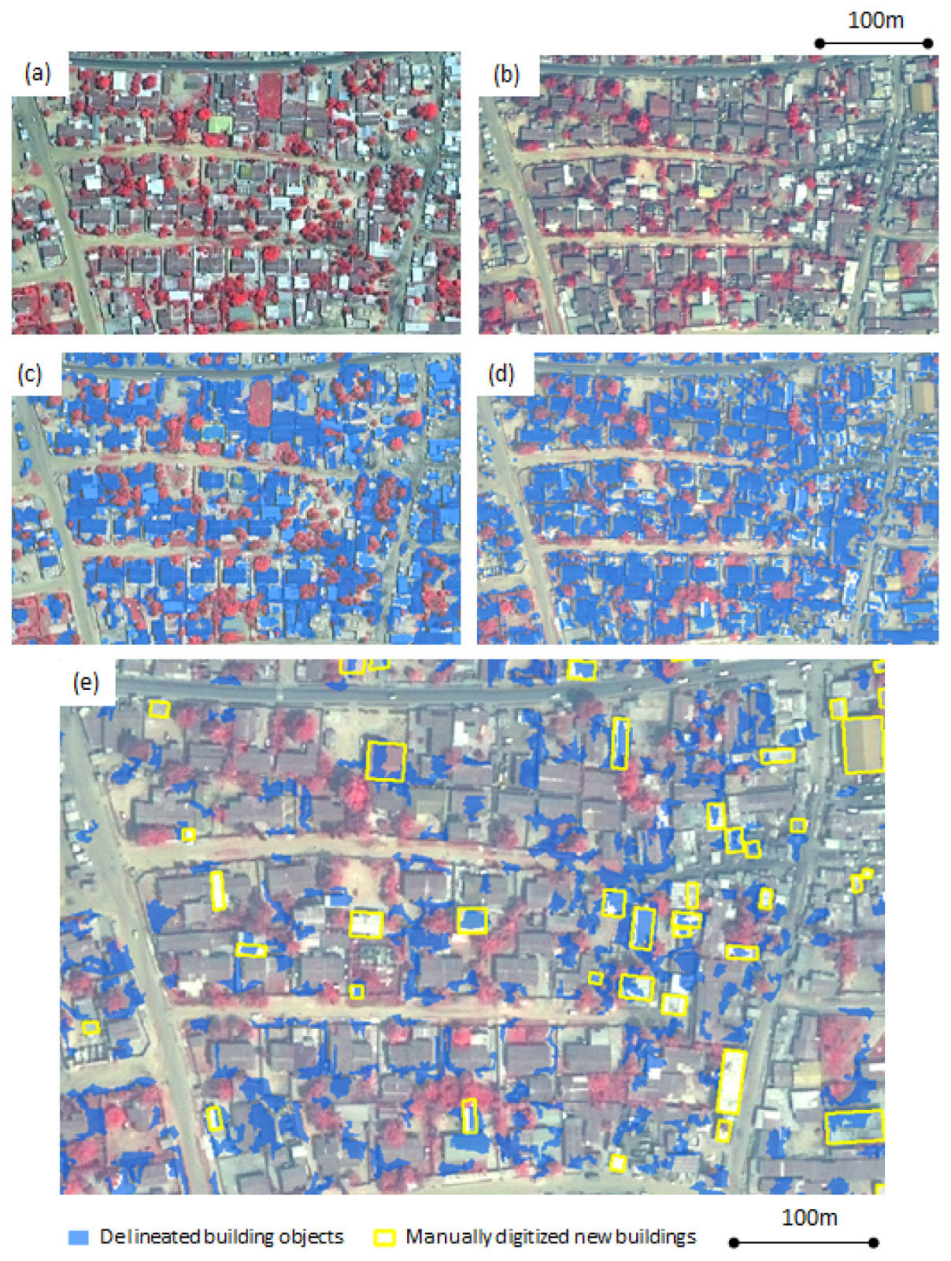
A subset of the delineated building map derived from the spectral/spatial contextual approach. **(a)** Pan-sharpened multispectral (PSMS) image of 2002. **(b)** PSMS image of 2010. **(c)** Objects classified as buildings in 2002 image (in blue). **(d)** Objects classified as buildings in 2010 image (in blue). **(e)** The delineated new buildings (in blue) using the post-classification comparison method. The actual new buildings are displayed in yellow. The over-classified building maps introduced errors into the final result.

False change detections were more prevalent with the post-classification comparison method since classification errors on either date led to false indications of change [1]. A substantial number of new building features were delineated correctly on the 2010 classification product, but many non-building changes were misclassified as new buildings. Figure 6 illustrates how the 2010 dataset had more non-building features classified as buildings. The post-classification comparison method was more susceptible to inconsistent sizes and shapes of delineated objects. It was difficult to produce comparable building objects between the two dates of imagery. These inconsistent objects eventually led to classification errors. Failure to correctly classify certain transition types between two dates caused errors in the results of bi-temporal layerstack approach. Omission errors tended to occur when the new buildings in Time 2 had limited spectral contrast to background features or darker rooftop materials in Time 1.

The aforementioned issues such as confusions between classes and object inconsistency influenced errors for both spectral contextual (Feature Analyst) and the true OBIA (Feature Extraction) approaches. The true OBIA approach produced higher commission errors due to the erroneous segments and objects. Not every object represented building shape accurately. The inconsistent delineation and classification of building objects affected this approach more than the spectral contextual approach. On the other hand, the errors in the spectral contextual approach results were mainly related to confusion between buildings and background features.

The two software packages require different means for creating training samples, which made it difficult to create consistent training sets. We attempted to keep track of which features were used as training data between two software and two dates of imagery. Also, it was very common in the true OBIA approach (Feature Extraction software) that building features were represented by multiple segmented sub-objects. Differences in training samples between the spectral contextual and the true OBIA approaches might have led to inconsistent objects and lower comparability.

Differences between the two sets of imagery likely contributed to change detection errors as well. Differences in illumination angles caused differential shading and cast shadows, and thus created classification errors that were manifested as building changes. A slightly different view angle and minor misregistration between two images did not seem to yield many change detection errors. The bi-temporal layerstack method seemed to handle these inconsistencies in imagery characteristics better and created more accurate results than the post-classification method. The spectral contextual approach results were relatively less affected by these inconsistences.

In terms of efficiency, it took about 23 h (0.66 h/km^2^) of user interaction and processing time to complete the post-classification comparison method and 15 h (0.23 h/km^2^) to process the bi-temporal layerstack with Feature Analyst software for the 35 km^2^ study area. For ENVI Feature Extraction, it took 61 h (1.74 h/km^2^) to complete the post-classification comparison method and 38 h (1.09 h/km^2^) to process the bi-temporal layerstack method. These times do not include the substantial amount of time for learning and experimenting with the software routines, and also, do not fully simulate production mode processing. Both Feature Analyst and ENVI Feature Extraction software provide effective interactive interfaces with preview functions that enabled rapid assessment of intermediate products at parameter setting phases. These allow users to generate products without spending excessive time or storage space on testing various segmentation and classification parameters. However, these image analysis software packages that utilize spectral, spatial and other contextual image information required intensive computation and long processing times, especially the object-based delineation approach. Also, substantial effort and time to select suitable training samples is required as we chose to utilize supervised classification approaches in both software packages. The post-classification comparison method in particular took longer time to process since the two building classification products were generated separately.

Upon manual editing (*i.e.*, heads-up digitizing) of a product derived from a semi-automated OBIA change process, a very accurate and efficiently derived map of the locations (but not necessarily the shapes) of new buildings was generated. As for many image processing endeavors, the most efficient means for generating a suitably accurate map of new buildings is often through such a hybrid process, rather than by generating the map strictly from manual interpretation and heads-up digitizing, or by semi-automated OBIA alone. Benchmarking was performed to compare the processing efficiency between identifying and digitizing new buildings based imagery, and editing an existing product for three image subsets totally 0.8 km^2^ in areal extent. The amount of time to generate a new building map through a completely manual and interactive procedure was 40 min (0.83 h/km^2^). Combining the estimate of 0.66 h/km^2^ for generating the image-derived map, using Feature Analyst and the post-classification comparison approach with an estimate of 19 min (0.40 h/km^2^) for the manual editing, yielded a total rate of 1.06 h/km^2^. While this suggests that the hybrid OBIA and manual editing approach took slightly longer than the manual, heads-up digitizing approach, we note that the OBIA processing time estimates are likely substantially over-estimated due to efficiencies associated with larger spatial extents and processing in a production mode. However, if precise representations of new building shapes and areas were a requirement, the shapes of most of the image-delineated objects would not be sufficiently accurate and would require complete manual delineation. We also note that assessing the accuracy of land cover change products derived from complete or partial manual interpretation of VHSR satellite imagery is extremely challenging, particularly in a developing country such as Ghana, as it is difficult to secure multi-temporal imagery that have even higher spatial resolution for similar time frames as the satellite data and impossible to go back in time to collect ground reference data.

## 5. Conclusions

This paper summarizes one of the first attempts at directly detecting and delineating new buildings by combining OBIA approaches with bi-temporal high resolution satellite data. Buildings constructed between 2002 and 2010 in Accra, Ghana were delineated and quantified; issues encountered during the change detection processing were identified. The primary utility of the OBIA-derived maps of new buildings in Accra is to support spatial analyses of LCLU and demographic change. The locations and densities of building changes provide information about densification and settlement of major cities within developing countries such as Accra. Bi-temporal layerstack classification method had less confusion between building and background objects and thus, generated more accurate results than the post-classification comparison method. The spectral/contextual approach was able to more reliably delineate buildings of various sizes compared to the true OBIA approach. However, at this stage of OBIA implementation, substantial manual editing of the OBIA-derived maps are required before reliable information on building changes can be extracted. Upon manual editing, we quantified 11,747 new buildings, meaning that approximately 340 new buildings were constructed per km^2^ from 2002 to 2010. A significant inverse relationship between new building density and housing quality index as well as qualitative patterns on resultant maps suggest that new building construction during the 2002 to 2010 period tended to be denser in low SES areas associated with slum-like conditions.

Future research will focus on attempts at improving the accuracy and efficiency of new building delineation. Incorporating thematic GIS layers (e.g., street or road maps) or LIDAR data into the building delineation process may improve final product accuracy (However, we note that acquisition of LIDAR data may be cost prohibitive in developing countries). Both Feature Analyst and ENVI Feature Extraction provide the option to include ancillary or multi-modal remote sensing data into the image classification process. These additional data layers have the potential to improve delineations and reduce classification confusion by maximizing differences between buildings and background features and materials. Creating more classes to differentiate various sizes of buildings and account for a greater range of roof material types during the training and classification phase should improve the object inconsistency issue and delineated map quality. Sequentially classifying smaller image subsets with similar building shape, size and material characteristics may also minimize inconsistency in object delineation and lower change detection errors. To further limit the effects of different image characteristics, image pairs with similar illumination condition and view angles are preferred, if available.

## Figures and Tables

**Table 1:** Number of Training Objects Used in Feature Analyst and Feature Extraction.

Classification Category	Post-Classification Comparison				Bi-Temporal Layerstack	
	2002		2010			
	FA*	FE*	FA*	FE*	FA*	FE*
Bright Roof	945	5450	927	3430	943	2391
Dark Roof	997	2798	1582	2554	1074	1186
Soil	496	1213	808	1103	543	637
Road	145	214	178	212	134	231
Shadow	185	1576	251	1090	44	384
Vegetation	496	1491	699	898	249	405
Cement	79	314	77	297	48	166
Old Vegetation	N/A	N/A	N/A	N/A	185	206
New Building	N/A	N/A	N/A	N/A	617	3892
Total	3343	13054	4522	9584	3837	9498

* FA: Feature Analyst. FE: Feature Extraction.

**Table 3:** Number of Delineated New Buildings.

Feature Delineation Approach	Number of New Buildings	
	Post-Classification Comparison	Bi-Temporal Layerstack
Spectral/Spatial Contextual	40,426	12,105
Object-based Image Analysis	45,439	48,208

**Table 4:** Feature Analyst Approach Results.

Parameter	Methods		
	Post-Classification Comparison		Bi-Temporal Layerstack
	2002	2010	
Kernel Pattern	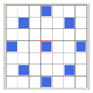 Bull’s Eye 3	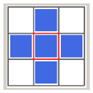 Bull’s Eye 3	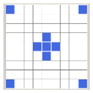 Bull’s Eye 1
Kernel Size (pixels)	7 × 7	3 × 3	9 × 9
Correctness (%)	34		59
Completeness (%)	42		37

**Table 5:** ENVI Feature Extraction Approach Results.

Parameter	Methods		
	Post-Classification Comparison		Bi-Temporal Layerstack
	2002	2010	
Scale Level	30	25	30
Merge Level	75	75	60
K Parameter	3	3	3
Correctness (%)	29		37
Completeness (%)	56		61.5
